# Editorial: Advances in non-alcoholic fatty liver disease therapeutics: Pathogenic mechanisms and targets

**DOI:** 10.3389/fphar.2022.1072353

**Published:** 2022-11-10

**Authors:** Ana Blas-García, Francisco Javier Cubero

**Affiliations:** ^1^ Department of Physiology, Faculty of Medicine, University of Valencia, Valencia, Spain; ^2^ FISABIO (Fundación para el Fomento de la Investigación Sanitaria y Biomédica de la Comunidad Valenciana), Valencia, Spain; ^3^ Centro de Investigación Biomédica en Red de Enfermedades Hepáticas y Digestivas (CIBEREHD), Madrid, Spain; ^4^ Department of Immunology, Ophthalmology and ENT, Complutense University School of Medicine, Madrid, Spain; ^5^ Instituto de Investigación Sanitaria Gregorio Marañón (IiSGM), Madrid, Spain

**Keywords:** NAFLD, liver, therapy, gut, microbiome, NASH

Non-alcoholic fatty liver disease (NAFLD) is a leading cause of advanced liver diseases worldwide and an increasing clinical and economic burden. As its complex pathophysiology and heterogeneity of disease phenotypes result in a lack of therapeutic approaches for NAFLD, efforts must continue to develop effective treatments for patients with advanced non-alcoholic steatohepatitis (NASH) and prevention methods for individuals at high risk of NAFLD and progressive liver disease.

As editors of this Research Topic, it was a joy to review a wide range of interesting manuscripts in relation to the novel targets and therapies for NAFLD. In total about 100 basic scientists and clinicians from different countries contributed 14 originals or review articles about current perspectives in novel targets and approaches for NAFLD resolution and prevention. In the current editorial we summarize the main contributions and take-home messages within each of the accepted articles.

Ma et al. analyzed the impact of empagliflozin, a novel type of sodium-glucose co-transporter two inhibitor, in a murine model of NAFLD. Transcriptomic analysis revealed that empagliflozin significantly decreased lipid synthesis and improved lipid metabolism, thereby enhancing triglyceride transfer, lipolysis and microsomal mitochondrial *β*-oxidation. Control trials with patients are needed with this already approved drug.

Osipova et al. reviewed the literature of the use of essential phospholipids (EPLs) rich in phosphatidylcholine (PCH) as a treatment option for fatty liver disease, demonstrating a robust clinical utility. This review nicely explored the potential molecular and metabolic pathways involved in the positive effects of PCH on steatosis regression.

The regulatory mechanisms of mutations in the lamin gene in lipid alterations in human disease is reviewed by Peng et al. Nuclear lamins, known as type 5 intermediate fibers, are composed of lamin A, lamin C, lamin B1, and lamin B2, which are encoded by *LMNA* and *LMNB* genes, respectively. Importantly, mutations in nuclear lamins not only participate in lipid disorders but also in different human diseases, such as lipodystrophy, metabolic-associated fatty liver disease, and dilated cardiomyopathy. Targeting nuclear lamins may be a potent therapeutic avenue for lipid metabolic disorders and human diseases in the future.

The study by Zeng et al. identified key genes linking NAFLD fibrosis and hepatocelullar carcinoma (HCC) through analysis and experimental verification using two GEO datasets. The results suggested that common differentially expressed genes (DEGs) were strongly associated with the glucocorticoid receptor pathway, regulation of transmembrane transporter activity, peroxisome, and proteoglycan biosynthetic process. Interestingly, the expression of nucleolar and spindle associated protein 1 (NUSAP1) was highly expressed in both human hepatic cell lines and NAFLD models at mRNA and protein level. These data revealed that modulation of NUSAP1 may be a therapeutic target for preventing NAFLD progression to liver cancer.

Fang et al. evaluated calcineurin 2 (RCAN2) expression in the liver of mice with hepatic steatosis and in the serum of NAFLD patients. They found that elevated serum RCAN2 levels were associated with an increased risk of NAFLD, suggesting that serum RCAN2, and especially the ratio between serum RCAN2 and liver enzymes, might be a candidate diagnostic marker for NAFLD.

Jian et al. showed that rifaximin greatly ameliorated hepatic steatosis, lobular inflammation, and fibrogenesis in mice with methionine and choline deficient diet-induced NASH *via* alteration of the gut microbiome. Rifaximin treatment may therefore be a promising approach for NASH therapy in humans.

Zhao et al. demonstrated that overexpression of fibroblast growth factor-9 (FGF9) in the liver of diet-induced obese mice inhibits the expression of genes involved in lipogenesis and increases the expression of genes involved in fatty acid oxidation, thereby reducing cellular lipid accumulation. Thus, targeting FGF9 might be exploited to treat NAFLD and metabolic syndrome.

The review by Zhang and Feng summarized the actual knowledge on the potential use of *Astragalus* polysaccharides (APS) for basic research, pharmacological development, and therapeutic applications in the management of NAFLD.

The pharmacological function and the underlying mechanisms of celastrol in the prevention and treatment of liver diseases including NAFLD, liver injury, and liver cancer was comprehensively reviewed by Li et al. Both preclinical and clinical studies are required to accelerate the clinical transformation of celastrol in a feasible treatment option.

Growing evidence suggests that thyroid hormone/thyroid hormone receptor (TH/TR) axis participates in hepatic metabolism, specifically in NAFLD, liver fibrosis, and in the progression of acute liver failure and alcoholic liver disease. Therefore, Tang et al. present pros and cons for the TH/TR axis as a prospecting target to cure hepatic diseases.

Huazhi Rougan Granule (HRG) is commonly used in the clinical treatment of NAFLD because it can reduce hepatic lipid deposition and regulate intestinal flora, thus exerting a protective mechanism. In a murine model, Liu et al. showed that the mechanism of HRG in the treatment of NAFLD through intestinal flora is mainly reflected in the biological process of gene function and related to infectious diseases, immune systems, and signal transduction pathways.

Lin et al. investigated the role of farnesoid X receptor (FXR) in the high-dose obeticholic acid (OCA)-induced hepatoxicity in a NAFLD mouse model. In their study, the authors showed that high-dose OCA induces cholesterol accumulation in livers *via* the upregulation of genes involved in cholesterol acquisition and the downregulation of genes regulating cholesterol degradation in liver, leading to the production of IL-1β and an FXR-mediated inflammatory response.

Chen et al. explored the causal relationship between NAFLD and inflammatory bowel disease (IBD) using a multivariable Mendelian randomization analysis. This study ruled out the causal relationship between these two pathologies, suggesting therapeutics targeting NAFLD might not work for IBD and *vice versa*.

Finally, the study by Li et al. analyzed the protective effect of oral administration of baicalein, a natural flavonoid with multiple biological activities, on NAFLD in high-fat diet murine models. Multi-omics analysis revealed that baicalein might affect lipid metabolism in liver via regulating the ecological structure of gut microbiota in NAFLD mice.

Altogether, this Research Topic deepens in novel mechanisms, drugs and targets for the treatment of NAFLD/NASH.

